# The Amyloid Precursor Protein of Alzheimer’s Disease Clusters at the Organelle/Microtubule Interface on Organelles that Bind Microtubules in an ATP Dependent Manner

**DOI:** 10.1371/journal.pone.0147808

**Published:** 2016-01-27

**Authors:** James W. Stevenson, Eliza A. Conaty, Rylie B. Walsh, Paul J. Poidomani, Colin M. Samoriski, Brianne J. Scollins, Joseph A. DeGiorgis

**Affiliations:** 1 Biology Department, Providence College, Providence, Rhode Island, United States of America; 2 Bell Center, Marine Biological Laboratory, Woods Hole, Massachusetts, United States of America; Torrey Pines Institute for Molecular Studies, UNITED STATES

## Abstract

The amyloid precursor protein (APP) is a causal agent in the pathogenesis of Alzheimer’s disease and is a transmembrane protein that associates with membrane-limited organelles. APP has been shown to co-purify through immunoprecipitation with a kinesin light chain suggesting that APP may act as a trailer hitch linking kinesin to its intercellular cargo, however this hypothesis has been challenged. Previously, we identified an mRNA transcript that encodes a squid homolog of human APP_770_. The human and squid isoforms share 60% sequence identity and 76% sequence similarity within the cytoplasmic domain and share 15 of the final 19 amino acids at the C-terminus establishing this highly conserved domain as a functionally import segment of the APP molecule. Here, we study the distribution of squid APP in extruded axoplasm as well as in a well-characterized reconstituted organelle/microtubule preparation from the squid giant axon in which organelles bind microtubules and move towards the microtubule plus-ends. We find that APP associates with microtubules by confocal microscopy and co-purifies with KI-washed axoplasmic organelles by sucrose density gradient fractionation. By electron microscopy, APP clusters at a single focal point on the surfaces of organelles and localizes to the organelle/microtubule interface. In addition, the association of APP-organelles with microtubules is an ATP dependent process suggesting that the APP-organelles contain a microtubule-based motor protein. Although a direct kinesin/APP association remains controversial, the distribution of APP at the organelle/microtubule interface strongly suggests that APP-organelles have an orientation and that APP like the Alzheimer’s protein tau has a microtubule-based function.

## Introduction

Alzheimer’s disease (AD) is a debilitating neurodegenerative disorder characterized by the loss of long-term memory, language degeneration, and cognitive impairment. This disease afflicts an estimated 26.6 million people worldwide and is predicted to reach an incidence of 100 million by 2050 [1]. Pathologically, AD is diagnosed by the presence of amyloid plaques in brain [2–5] that contain Aβ a peptide fragment of the amyloid precursor protein [6]. While most forms of AD are considered sporadic, mutations in APP cause heritable forms of this disorder, thus establishing a causal role for this protein in AD pathogenesis [7–10].

It is well established that APP contains a single transmembrane domain that spans the lipid bilayer of membrane-limited organelles [11]. APP is cleaved through two distinct enzymatic pathways, one that yields the pathogenic Aβ fragment and another that cleaves within the Aβ domain to produce a non-pathogenic physiology [12]. While the N-terminal of APP resides within the organelle lumen, the C-terminal extends into the neuronal cytoplasm [13]. APP-associated organelles are transported through processes of fast axonal transport [14] and immunoprecipitation studies have shown that APP co-purifies with a kinesin light chain [15]. These findings suggest that APP may link kinesin to its cellular cargo, however this theory is under debate [16].

For many years, we have been interested in motor-driven organelle movements and have studied these processes in the squid giant axon, the model in which the first direct observations of axonal transport were made and the system in which conventional kinesin (Kinesin-1) the founding member of the kinesin motor family was discovered [17–20]. To identify other motors and to obtain genetic information on squid, we undertook an expressed sequence tag project by single-pass sequencing randomly selected mRNAs of the squid stellate ganglia. Along with finding a variety of motors, we identified a single transcript that encodes a squid homolog of human APP [21]. By immunoblot we find that antibodies to the C-terminal of human APP recognize a single band in squid axoplasm at the predicted molecular weight of the squid APP protein, thus demonstrating that APP is present in the squid giant axon [22].

Within the intact squid axon as well as in extruded axoplasm, organelles move bidirectionally along microtubules, and in reconstituted motility assays isolated organelles move towards the microtubule plus-ends [23–26]. Here, we set out to determine the distribution of APP in this well characterized system. Surprisingly, we find that APP clusters at a single focal point on the organelle surface and localizes to the organelle/microtubule interface. In addition, these APP-organelles associate with microtubules in an ATP dependent manner a biochemical characteristic of microtubule-based motors, thus it is likely that APP and a molecular motor coexist at the juncture between the cargo and the intercellular road.

## Materials and Methods

Live North Atlantic Long-Finned Squid *Doryteuthis pealeii* (formerly *Lologo p*.) were obtained from the Marine Resources Department, Marine Biological Laboratory, Woods Hole, Massachusetts. Squid giant axons were dissected under fresh, well-oxygenated, running seawater and used immediately in the procedures outlined below.

### Immuno-fluorescent labeling of APP and tubulin in extruded axoplasm

Axoplasm was extruded from freshly dissected squid axons onto 24X60 mm No. 1.5 cover glass and blocked for 1 hour in General Tubulin Buffer (80 mM PIPES (pH 6.9), 1 mM EGTA, 1 mM MgCl_2_) containing 10 mg/ml BSA and 20 μm paclitaxel (Sigma Aldrich, St Louis MO). Samples were immuno-labeled for APP and microtubules by incubating axoplasm in a 1:200 dilution each of a rabbit anti-human APP (Invitrogen Inc., Grand Island, NY [product # 51–2700]) and mouse anti-tubulin antibody (Sigma Aldrich, St Louis, MO [product # T9026]) in blocking solution for 1 hour. The APP antibody termed CT695 was raised against the final 22 amino acids of the C-terminus of human APP. This epitope is conserved between human and mouse homologs (100%) and shares 15 identical (68%) and 19 similar (86%) amino acid residues with squid APP. Samples were washed in blocking buffer 3 x 10 min and then treated with 1:200 donkey anti-rabbit Alex Flour^®^ 488 and donkey anti-mouse Alex Flour^®^ 594 fluorescent secondary antibodies diluted in blocking solution (Jackson ImmunoResearch Laboratories, Inc., West Grove, PA). Samples were mounted on glass slides with Vectashield mounting media (Vector Laboratories, Inc. Burlingame, CA) and imaged with an inverted Zeiss 710 Laser Scanning Confocal Microscope (Carl Zeiss, Microscopy, LLC, Thornwood, NY).

### Isolation of KI-washed axoplasmic organelles

KI-washed axoplasmic organelles were obtained as previously described [25,26]. Briefly, approximately 50 μl of axoplasm was extruded from 10 freshly dissected squid axons into a 50 μl drop of 1/2X buffer (10 mM HEPES–KOH (pH 7.2), 175 mM L-aspartic acid, 65 mM taurine, 85 mM betaine, 25 mM glycine, 6.5 mM MgCl_2_, 5 mM EGTA, 0.5 mM D-glucose, 1.5 mM CaCl_2_) containing 1 mM DTT and protease inhibitors (10 mM each of benzamidine, leupeptin, pepstatin A, aprotinin, and phenanthroline). Potassium iodide (3 M in 1/2X buffer) was added to the sample to a final concentration of 600 mM to dissociate the cytoskeleton. The resulting sample was triturated 30 times with a yellow tipped pipette and then placed on ice for 10 min. The sample was diluted 1:1 in 1/2X buffer and layered onto a three step sucrose gradient consisting of 100 μl of 45%, 200 μl of 15%, and 100 μl of 12% sucrose in 1/2X buffer layered within a Beckman 0.7 ml ultracentrifuge tube (Beckman Coulter Inc., Indianapolis, IN). The sample was centrifuged at 100,000 X g in a Beckman 52Ti swinging bucket rotor at 35,000 RPM for 90 min at 4°C. The supernatant was drawn off the gradient surface by pipette and the 15% sucrose layer containing the KI-washed organelle isolate was removed by side puncture using a needle and syringe. The supernatant and KI-washed organelles were used for immunoblots. Organelles were also used for immunocytochemistry and reconstituted organelle/microtubule preparations.

### SDS-PAGE and immunoblot analysis of APP in axoplasmic fractions

Sucrose density gradient samples of axoplasmic supernatants and isolated KI-washed organelles (20 μl each) were run on 10% SDS polyacrylamide gels along with a broad range molecular weight marker (Bio-Rad Inc. Carlsbad, CA). Parallel gel sets were either Coomassie stained or transferred to nitrocellulose. Transfers were incubated in a blocking solution for 1 hour (5% Carnation instant milk, 0.2% Tween-20 in TBS). Blots were incubated in a 1:1,000 dilution of rabbit anti-human APP antibody in blocking solution for 2 hours followed by 3 X 10 min wash in blocking solution alone. Blots were incubated in a peroxidase conjugated secondary antibody (Amersham, GE Healthcare, Waukesha, WI) at 1:5,000 for 1 hour in blocking solution and then washed 3 X 10 min in Tris Buffered Saline. Blots were soaked in a chemiluminescent developer and sheet film exposed to the membrane surface. Exposed film was developed and scanned to obtain a digital image of the immunoblot. Images were color inverted to obtain black immuno-bands on a white background.

### Immunolabeling of isolated KI-washed axoplasmic organelles for TEM

Formvar carbon-coated EM grids were placed on 30 μl drops of isolated KI-washed axoplasmic organelles for 2 min. EM grids were floated sample side down on 50 μl drops of blocking solution (10 mg/ml BSA in PBS) for 1 hour. Samples were transferred to drops of anti-APP antibody diluted 1:200 in blocking solution for 1 hour, washed 3 x 10 min in blocking solution alone, and incubated in 12 nm colloidal gold conjugated secondary antibodies in blocking solution (1:200) for 1 hour (Jackson ImmunoResearch Laboratories, Inc., West Grove, PA). Samples were washed in PBS 3 X 10 min followed by incubation on drops of 1% uranyl acetate (UA) in water for 2 min. Excess UA was removed and samples were air dried before imaging. The samples were imaged with a Jeol CX200 electron microscope (Jeol Ltd., Peabody, MA) with an AMT camera (Advanced Microscopy Techniques, Corp. Woburn, MA) and gold particle distribution observed and photographed at 50,000X.

### Preparation and immunolabeling of reconstituted organelle/microtubule complexes in the presence and absence of ATP

Lyophilized bovine tubulin (Cytoskeleton Inc. Denver, CO) was resuspended in General Tubulin Buffer (80 mM PIPES (pH 6.9), 1 mM EGTA, 1 mM MgCl_2_) containing 5% glycerol and 1 mM GTP to a final tubulin concentration of 0.5 mg/ml and snap frozen in 5 μl aliquots for storage. For experiments, aliquots were thawed and placed at 35°C for 20 min. Warm General Tubulin Buffer (100 μl) containing 20 μM paclitaxel (Sigma Aldrich, St Louis, MO) was added to aliquots and tubulin samples incubated at room temperature overnight to promote microtubule polymerization. The resulting microtubule solution was added at a 1:1 ratio by volume to isolated KI-washed axoplasmic organelles supplemented with 20 μm paclitaxel in the presence and absence of 10 mM ATP final concentration and incubated at room temperature for 30 min. Formvar carbon-coated EM grids were incubated on 20 μl droplets of organelle/microtubule samples for 1 min, washed 3 x 5 min in PBS containing 20 μm paclitaxel, and immunolabeled for APP as outlines above (all solutions contained 20 μm paclitaxel to preserve microtubule integrity). After immunolabeling, samples were stained in 1% uranyl acetate in water. Samples with and without ATP were imaged at 50,000 X by transmission electron microscopy. The association of KI-washed APP-organelles with exogenous microtubules in the presence and absence of ATP were analyzed as outlined below.

### Immunolabeling of axoplasmic touch preparations

Axoplasm for squid giant axons was extruded onto parafilm and formvar carbon-coated copper grids touched lightly to the axoplasm to obtain a thin layer of axoplasmic components on the formvar surface. Samples were immuno-labeled for APP using 12 nm gold secondary antibodies in the presence of 20 μm paclitaxel as outlined above. EM grids were photographed and APP distribution analyzed.

## Results

### APP is bound to microtubules in extruded axoplasm by confocal microscopy

**Fig 1:**
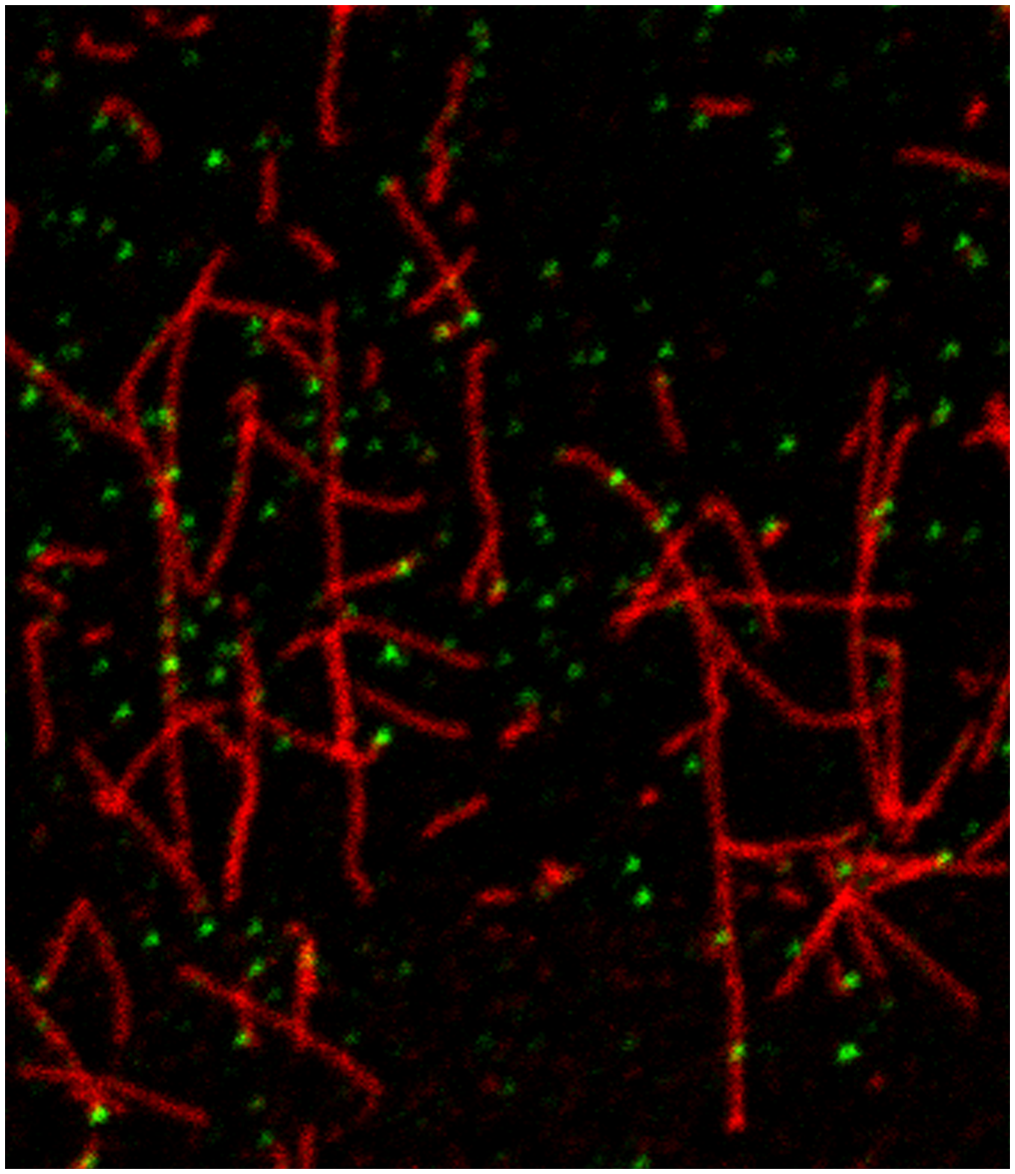
Immunofluorescence microscopy of APP and microtubules in extruded axoplasm. Axoplasm from the squid giant axon was extruded onto glass coverslips and fluorescently labeled with antibodies to the amyloid precursor protein (green) and tubulin (red). APP appears as punctate structures attached or unattached to microtubules lying along the glass surface.

In previous experiments, we found an amyloid precursor protein transcript in squid that expresses a protein product in the squid giant axon as demonstrated by immunoblot [22]. To determine whether this APP could be detected in the axon by immunofluorescence, axoplasm of the squid giant axon was extruded onto glass cover slips, stabilized with taxol, and labeled with antibodies raised against APP and tubulin (Fig 1). By confocal microscopy, microtubules appear as thin filaments that intersect one another and stretch out along the glass surface. APP labeling appears as discrete puncta that often associate with tubulin filaments. Some puncta are found on the top of the microtubule, while others appear along the microtubule edge. In some cases, puncta are found in regions distinct from the microtubule domain and may be bound to the glass surface or to other undetected cytoplasmic elements. The amount of APP labeling indicates that this protein is abundant in the axon and the intensity of the labeling suggests that the puncta are composed of multiple APP proteins.

### APP co-purifies with isolated axoplasmic organelles by sucrose density gradient fractionation

**Fig 2:**
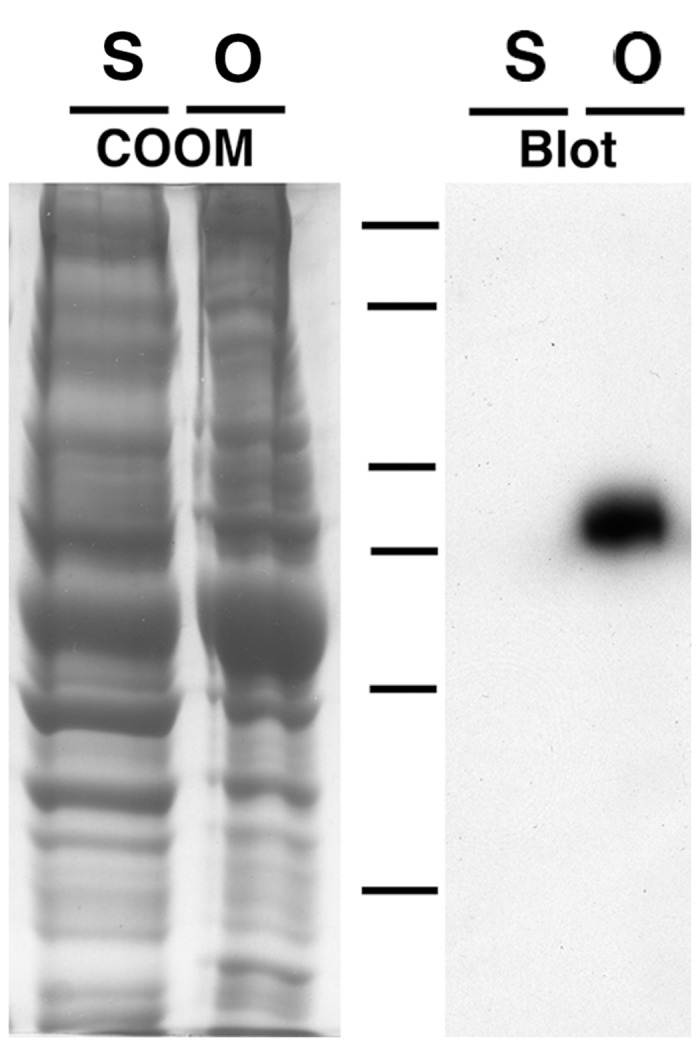
SDS-PAGE and immunoblot analysis of sucrose density gradient axoplasmic fractionations. Total squid axoplasm was treated with 600 mM KI and centrifuged over a three-step sucrose density gradient. The resulting supernatant and organelle fractions were used for Coomassie stained gels (Coom, S, and O) and immunoblots (Blot, S, and O) probed with a C-terminal anti-APP polyclonal antibody (see materials and methods) and detected through chemiluminescence. Dashes indicate molecular weight markers: 250, 150, 100, 75, 50, 37 kDa.

Like human APP, the amino acid sequence of the APP in squid contains a single transmembrane domain, suggesting that this protein like human APP spans the membranes of organelles. It has been shown in reconstituted motility assays that organelles isolated from the squid giant axon move towards the plus-ends of microtubules in an ATP dependent fashion and that a Kinesin-3 is present on their surfaces [25,26]. In order to determine whether these organelles also contain APP, organelles were purified in the presence of 600 mM KI using an established sucrose density gradient protocol developed for investigating microtubule-based transport [25,26]. By immunoblot, we find that APP co-purifies with KI-washed axoplasmic organelles, while an axoplasmic supernatant is void of detectable levels of APP protein (Fig 2). APP appears to be abundant in the organelle fraction based on the thickness and intensity of APP bands obtained in blots. The band detected by anti-APP is in the molecular weight range predicted from the full-length squid APP amino acid sequence of ~70.3 kDa (612 aa [accession no. DQ913735]). The anti-APP antibody recognizes only a single band in organelle fractions. No other bands are detected suggesting that APP cleavage products or splice variants are not present within the axon. These experiments also demonstrate that the antibody raised against human APP is a useful tool for studying APP in the squid system.

### APP clusters at a single focal point on the surfaces of KI-washed axon organelles

**Fig 3:**
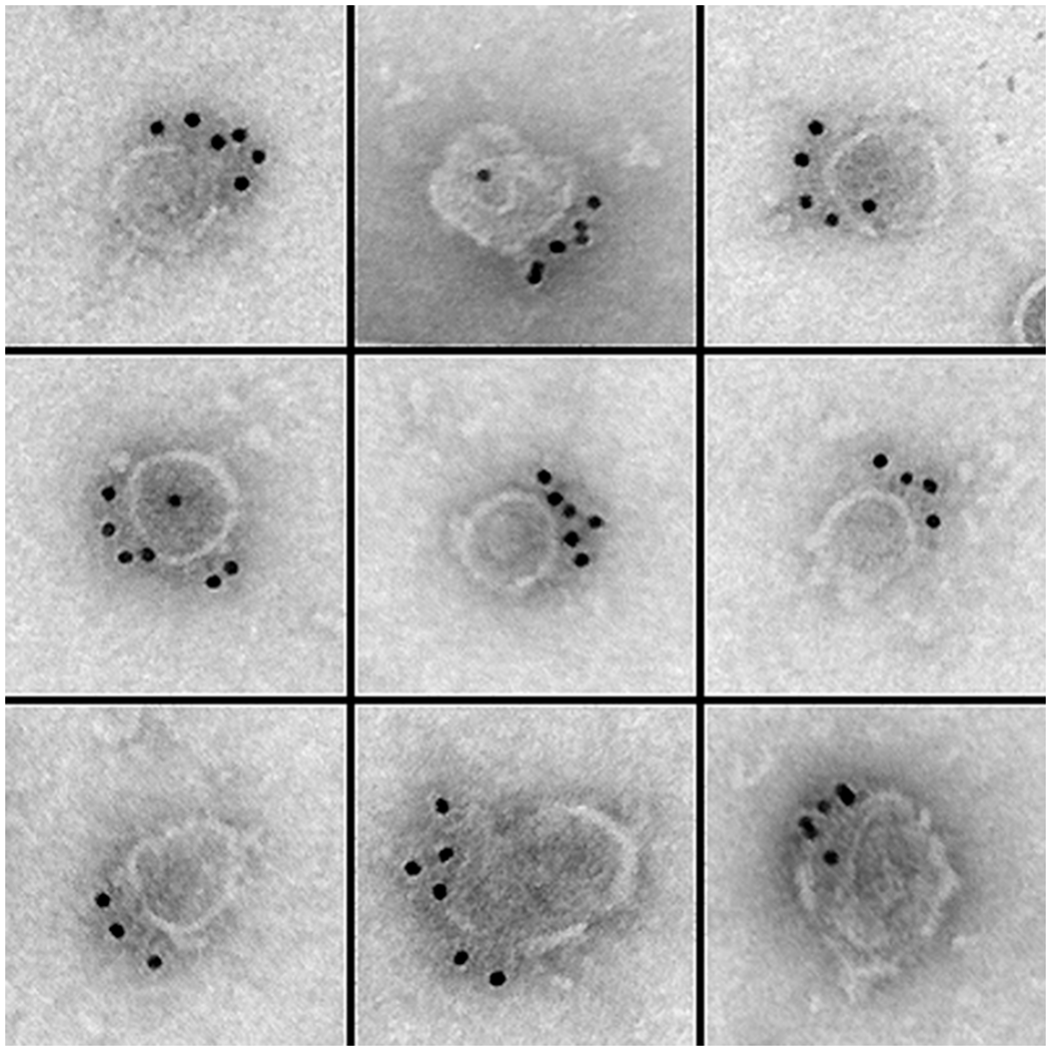
Immuno-localization of APP to the surfaces of isolated axoplasmic organelles. An APP antibody raised against the C-terminal of human APP was used to determine whether APP is directly bound to isolated axoplasmic organelles. Using a colloidal gold secondary antibody, gold particles were found to cluster on the surfaces of axoplasmic organelles through transmission electron microscopy.

To determine whether the co-purification of APP with KI-washed organelles in sucrose gradients is due to a direct association, formvar carbon-coated EM grids were incubated on droplets of the organelle fraction, labeled for APP, and stained for electron microscopy. As a negative control, duplicate samples were labeled with the secondary antibody only. By EM, fields of organelles were found along the formvar surface and many organelles were decorated by APP antibodies and colloidal gold. Surprisingly, the gold particles appear to cluster on the surfaces of organelles and appear to localize at single focal point on the organelle surface (Fig 3).

In the electron microscope, fields of APP labeled and control organelles were photographed at 50,000 X (four experiments, 50 images each) and the number of labeled organelles determined as a percentage of the total. While 72% of organelles were labeled for APP with a least one gold particle (n = 1,652), only 3% of organelles were label with secondary antibody only (n = 1,134). APP labeled organelles appear as flattened, round, membranated structures. Since the APP antibody was raised against the C-terminus of human APP the positive label on organelles demonstrates that the C-terminus of squid APP, like that in human, extends from the organelle into the neuronal cytoplasm [11].

To investigate APP clustering and distribution of APP relative to the organelle a line was drawn through the center of each organelle to create two equal hemispheres (set of 50 fields). The line angle was drawn through the organelle in such a manner to obtain a maximum number of particles within a single hemisphere, while maintaining a line that contains the center point of the organelle (fixed point of the circle). Particles were counted within the two hemispheres. Of 273 organelles, 78% contained particles only in a single hemisphere and of organelles that contain particles in both hemispheres (n = 60), 92% of the particles were found in the particle dominant sector. To determine whether gold particle clustering was due to secondary antibody clumping, the secondary antibody was diluted 1:1,000 in PBS and formvar-coated grids incubated on the diluted secondary conjugate. Fields of gold particles were imaged and singletons and gold clumps counted. Gold clumps were defined as two or more particles closer than one particle apart (12 nm). Of 1,041 particles only 62 gold clumps (two or more particles in close proximity) were observed (0.60%).

The finding that APP is clustered on the organelle surface is unpredicted and to the best of our knowledge is the first direct evidence of full-length APP clustering on membrane surfaces. In addition, the diameters of labeled organelles were measured along their narrowest axis and determined to be 97 ± 3 nm (n = 300) the same size as those previously shown to exhibit attached Kinesin-3 [26].

### APP-bound axoplasmic organelles associated with microtubules in an ATP dependent fashion

The finding that APP clusters and localizes to the surfaces of axoplasmic organelles led us to wonder whether these same organelles could bind to microtubules in the absence of ATP, a biochemical characteristic of microtubule-based motor proteins. Both conventional kinesin and cytoplasmic dynein were purified through microtubule affinity in the absence of ATP [20,27–31]. These motors move along microtubules with physiological levels of ATP and release from filaments at High ATP levels. Squid axoplasmic organelles also possess this biochemical characteristic demonstrating that these organelles contain microtubule-based motors on their surfaces [25].

**Fig 4:**
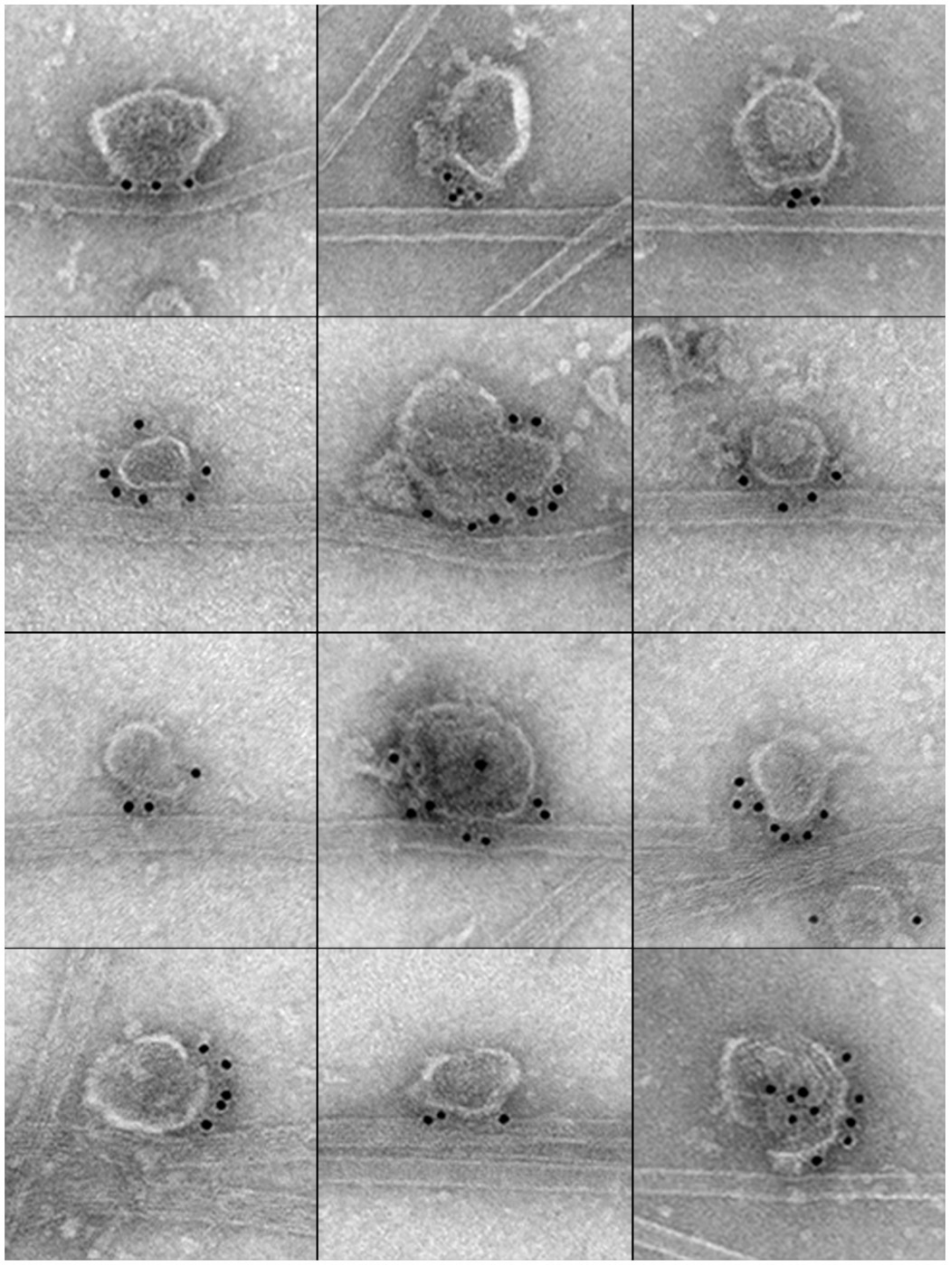
APP distribution in reconstituted organelle/microtubule complexes. Isolated KI-organelles were added to paclitaxel stabilized microtubules at a 1:1 ratio volume:volume and incubated at room temperature for 30 min to form reconstituted organelle/MT complexes. Formvar carbon coated copper EM grids were placed on 30 μl drops of complexes to adhere complexes to the grid surface. Complexes were labeled for APP using an Anti-APP primary antibody and a 12 nm colloidal gold conjugated secondary antibody. Images were taken at 50,000X with a Jeol 200CX transmission electron microscope and an AMT digital camera. Representative photos are presented in the montage. Gold particles appear in large foci on each organelle forming a cluster along the organelle surface, while the adjacent side of the organelle remains free of gold particles.

Here, purified KI-washed organelles were added to taxol stabilized bovine microtubules in the presence and absence of 10 mM ATP and the mixture incubated at room temperature for 30 minutes (Fig 4). EM grids were incubated of droplets of the samples and immunolabeled for APP. Samples in the presence and absence of ATP were photographed by transmission electron microscopy at 50,000X and resulting images analyzed. In samples that lack ATP, organelles attached to microtubules at a frequency of 1 APP-organelle per 78 microns in microtubule length, while APP-organelles were found mostly on formvar surfaces unattached to microtubule filaments (1:872 nm) when ATP was present. In addition, APP is found on 67% of the vesicles found associated with the microtubules. Further analysis of APP-organelle/microtubule complexes showed that 92% of the APP labeled distributed to the organelle/microtubule interface.

### APP localizes to the organelle/microtubule interface in axoplasmic spreads

**Fig 5:**
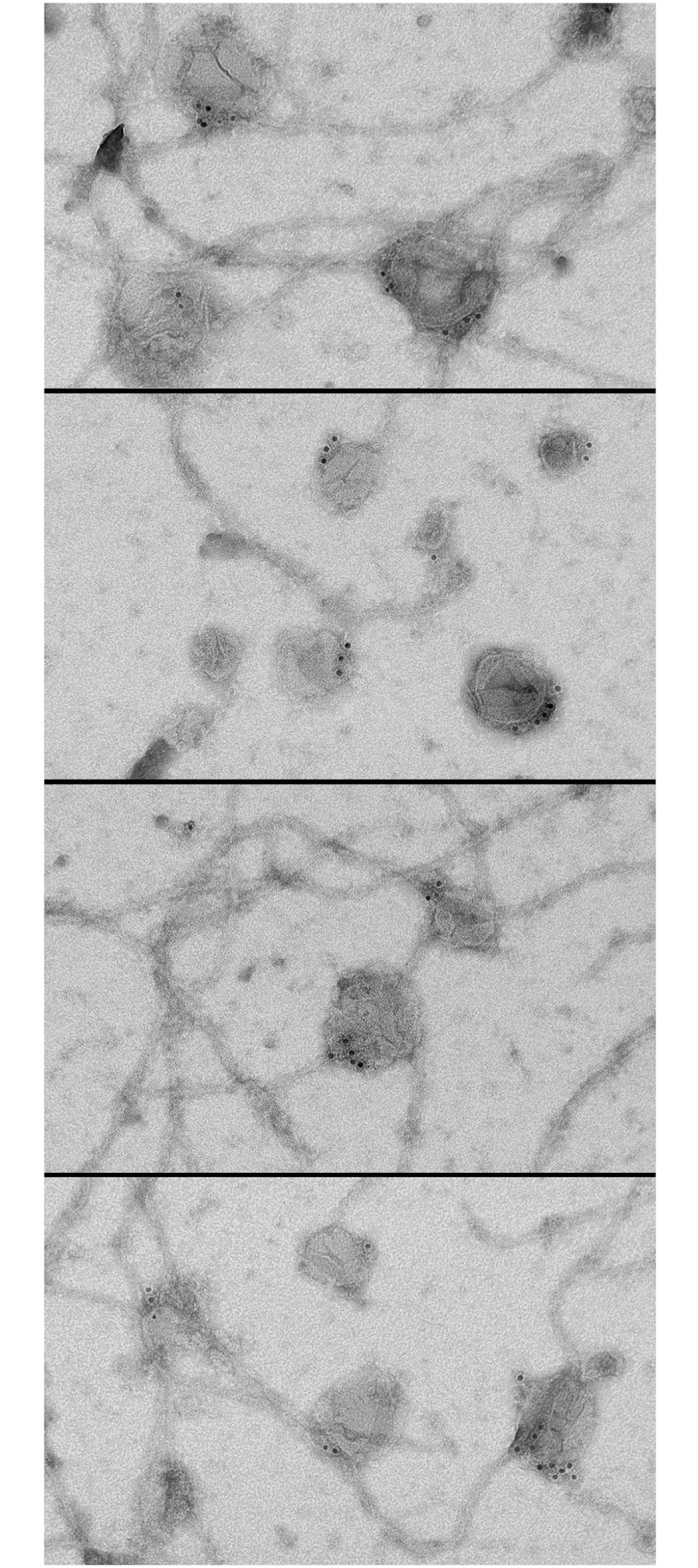
Immuno-gold labeling of axoplasmic touch preparations. Total axoplasm was extruded from a freshly dissected squid giant axon onto parafilm and formvar carbon-coated copper EM grids touch to the axoplasm to obtain a thin layer of axoplasmic components on the formvar surface. The sample was labeled with immuno-gold for the presence of APP using a C-terminal APP antibody.

To investigate the distribution of APP in axoplasm at the ultrastructural level, EM grids were touched to the surface of extruded axoplasm to obtain a thin layer of cytoplasmic elements on the grid surface and the sample immunolabeled for APP. On grids where axoplasm was thin enough to recognize cellular structures, microtubules, actin, mitochondria, organelles and other cytoskeletal elements could be seen by negative stain. Gold particles could be seen on organelle surfaces and labeling appears similar to APP labeling on isolated KI-treated organelles or reconstituted organelle/microtubule complexes. As a negative control additional grids were labeled with secondary antibody only. Interestingly, the distribution of gold appeared on organelles at the organelle/microtubule interface (Fig 5) as well as on organelles free from microtubules on the formvar surface. Not all free or microtubule-bound organelles contain label.

We determined the density of particle distribution on organelles, along microtubules and in the background of axoplasmic spreads. Label density was also determined for the background as well as for control samples in which the axoplasm was labeled with secondary only. The density of gold particles on microtubules under the organelle is 163 ± 14.6 compared to microtubule domains that lack the presence of an organelle that label at a density of 3.8 ± 0.6 per 100 μm^2^. Labeling for secondary only was low at organelle/microtubule interfaces, on microtubules alone as well as in the background with the highest labeling of 0.8 ± 0.2 on background surfaces (Table 1).

**Table 1:** Distribution of APP label in axoplasmic spreads (particles/μm^2^).

	Anti-APP Antibody (three experiments)	Secondary Only (three experiments)
Organelle/Microtubule Complexes	163 ± 14.6 (n = 562)	0 (n = 502)
Microtubules	3.8 ± 0.6 (100 μm^2^)	0.4 ± 0.2 (100 μm^2^)
Background	1.3 ± 0.3	0.8 ± 0.2

To determine whether APP distribution preferentially localized to the organelle/microtubule interface we established an interface zone. We reasoned that a colloidal gold particle within 25 nm of the interface could be attached to an epitope at that interface given the overall potential length of the APP cytoplasmic domain as well as the length of the primary antibody and secondary antibodies and the gold particle. Particles outside of the interface zone, were not considered to be attached to an epitope at the interface. In samples labeled for APP, 61% of the gold particles were within the interface zone, while 39% of the gold particles were found on the organelle, but were considered to label APP outside of the interface. However, 89% of labeled organelles that were attached to microtubules contained at least one gold particle at the interface.

**Fig 6:**
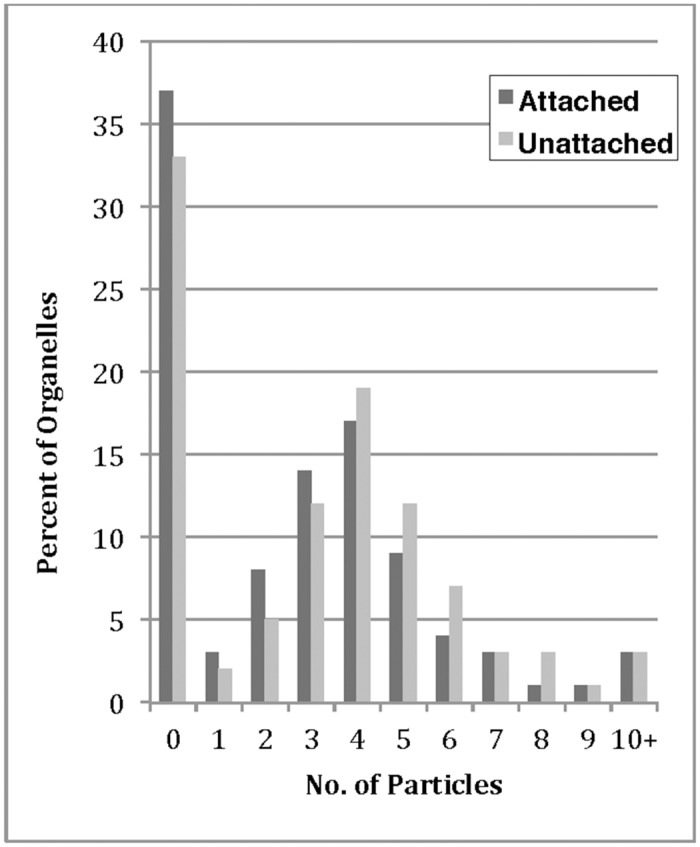
Frequency histogram of APP-gold labeling on free organelles and on organelles attached to microtubules in axoplasmic spreads. The number of gold particles on organelles attached to microtubules and organelles free from microtubules in the cytoplasm were counted on axoplasmic touch preparations. The number of gold particles on each organelle was plotted against the percentage of total organelles for both attached and unattached categories.

The number of particles on each organelle either attached or unattached to a microtubule filaments were counted. The percentage of organelles that lacked gold decoration was found to be 37% and 33% for attached and unattached respectively. For labeled organelles in both attached and unattached categories, the highest percentage of particle frequency was four. Some organelles contained more than 10 particles (Fig 6). It should be noted that immunogold labeling likely underrepresents the number of APP molecules present. Labeling is dependent on the labeling efficiency in terms of the antibodies ability to bind the antigen. The highly conserve antigenic site of APP has been suggested to have multiple binding partners including kinesin motors and this binding may mask antibody recognition. In addition, the organelles being labeled are in direct contact with the surface of formvar coated EM grids and hence large portions of the organelle surface area was blocked from antibody detection.

## Discussion

While it is well established that the amyloid precursor protein plays a causal role in Alzheimer’s disease the endogenous wild-type function of this molecule and the mechanisms through which it leads to disease is poorly understood. In humans, mice, and other mammals APP is a member of a small family of proteins including APP and the amyloid precursor-like proteins APLP1 and APLP2 [11,32–34]. Along with similarities in sequence and domain structure these proteins appear to be processed in a similar manner as each exhibit alpha, beta, gamma, and epsilon-like cleavage to yield similar proteolytic fragments [35]. However, of the three only APP has been implicated in neurological disease.

Knockout experiments aimed at determining function, despite the extensive effort, have been somewhat disappointing as individual knockouts for all three genes develop only mild phenotypes. The APP knockout exhibits a 15–20% weight reduction and a slight decrease in locomotor activity [36], while single knockouts for APLP1 and APLP2 have been largely uninformative [37,38]. A highlight of this work has been that APP and APLP1 have been shown to have overlapping functions with APLP2 as double knockouts of APP/APLP2 and APLP1/APLP2 exhibit early prenatal death clearly demonstrating an essential function for these proteins [37,38] and the redundancy of APP and APLP2 function indicates that a down regulation of APP could be a viable treatment for AD as APLP2 could substitute for the vital APP functions without contributing to disease.

In investigations of APP distribution, localization of APP suggests a ubiquitous role as APP is found in a wide variety of tissue types including brain, spinal cord, retina, spleen, muscle, kidney, lung, thymus, pancreas, and skin [39, 40]. Within neurons, APP localizes to the cell body and Golgi, as well as to axons, dendrites, and pre- and postsynaptic terminals [41–45]. It has been demonstrated that APP is transported to the cell surface in Golgi derived vesicles where it is incorporated into the cell membrane and is then recycled through clatherin mediated endocytosis [43]. APP has been found to associate with synaptic vesicles in the presynaptic terminal and in neurons in which APP and APLP2 is depleted there is a decrease in glutamate release suggesting that APP and APLP2 may be involved in synaptic transmission [46,47]. Many studies suggest that APP acts as a trophic factor involved in regulating cell proliferation, neuronal development, synaptogenesis, synaptic integrity, synaptic plasticity, and homeostasis as well as in cell adhesion [48–53].

The primary structure also suggests that APP is multi-functional. The orientation of APP relative to the vesicle surface is such that the N-terminal of APP resides within the vesicle lumen, while the single transmembrane domain spans the vesicle membrane and the C-terminal extends into the cellular cytoplasm. The N-terminal domain is characterized by a series of motifs including a copper-binding domain, a growth-factor-like-domain (GFLD) and an acidic domain (AcD) each of which likely convey APP activities [11,13,54]. Indeed, APP has been shown to bind copper and has been attributed to a role in copper homeostasis [55].

Adding to this complexity, intriguingly the C-terminal contains a highly conserved sequence of unknown function yet this high conservation suggests an important functional domain that may be responsible for the primary activity of APP and could provide a target for disease control or prevention. Many studies establish that APP is transported within neurons [56–59] and some studies suggest that it may play an active role in the transport process. Studies in which axons are constricted by ligature show a buildup of APP on the proximal side of the construction demonstrating that APP is in transit in the anterograde direction [60] and mutations in APP have been shown to disrupt axonal transport in *Drosophila* [61]. In other studies, latex beads coated with the conserved sequence and injected into axons move away from the cell body at rates of fast axonal transport suggesting that this sequence picks up a motor to propel the beads down the axon [22] and in immunoprecipitation studies APP co-precipitates with the Kinesin-1 light chain in mouse brain and sciatic nerve indicating a link between APP and the Kinesin-1 motor [15].

In the current study, we find that APP associates with a well-characterized set of squid axoplasmic organelles that move towards the plus ends of microtubules. These organelles bind microtubules in the absence of ATP and dissociate from filaments in its presence a biochemical characteristic of microtubule-based motors [20,25]. We find that APP clusters on the organelle surface at a focal point that localizes to the organelle/microtubule interface where molecular motors involved in microtubule-based transport must necessarily lie. These findings are in agreement with a role for APP in transport processes and indeed that APP may be a potential link between the motor and its cargo. However, the immunoprecipitation studies mentioned above have been strongly refuted [16] and evidence presented in the current study along with results from our previous work [26] argue against a connection between APP and Kinesin-1 at least in the squid system, although it is concurrent with an association between APP and another kinesin family member.

In the elegant experiments that led to the watershed discovery of conventional kinesin (Kinesin-1) and the kinesin motor family, purified squid Kinesin-1 was shown to facilitate microtubule gliding along glass coverslips and to move latex beads towards the plus-ends of microtubules [20] and its role attributed to moving organelles along microtubules in vivo. This protein was shown to be highly abundant in the squid giant axon, however little evidence linked conventional kinesin to organelles or to organelle trafficking. In our efforts to study microtubule-based transport in the squid system, we found that organelles isolated from squid axoplasm in the presence of 600 mM KI lacked Kinesin-1 despite the fact that they retained their ability to move towards microtubule plus-ends. In contrast, we found an abundance of a squid Kinesin-3 that co-purified with KI-washed organelles through sucrose density gradient fractionation [26]. Antibodies to the Kinesin-3 protein decorated the surfaces of these organelles and localized to the organelle/microtubule interface in reconstituted systems as well as in extruded axoplasm. In addition, antibodies to Kinesin-3 inhibited all plus-end microtubule-based movements of isolated organelles in reconstituted systems suggesting that Kinesin-3 is the only active motor in the KI-washed organelle fraction. Therefore the finding in this study that APP associates with KI-washed organelles that bind to microtubules in an ATP dependent manner strongly suggests that it is APP and Kinesin-3 that are found on the same organelle surface.

Our data does not rule out the possibility that Kinesin-1 associates with APP or that Kinesin-1 in squid is involved in transport. The high concentration of KI could strip kinesin motors from APP or the organelle surface. Indeed, the addition of KI to axoplasmic organelles abolishes minus-end directed movements along microtubules in reconstituted motility assays [25]. Minus-end directed movement is reestablished by adding a non-KI treated axoplasmic supernatant suggesting that dynein or a dynein co-factor is removed or inhibited by KI treatment [25]. However, the finding that Kinesin-3 remains bound to organelles even in the presence of high KI concentration demonstrates that this kinesin is tightly linked directly or indirectly to the organelle surface. In Kinesin-3 experiments organelles contained one or two gold labels a finding consistent with there being only a single Kinesin-3 on each organelle surface. If Kinesin-3 and APP are bound to one another, the purification of Kinesin-3 should yield APP even under stringent biochemical conditions.

The finding that APP clusters at a single focal point on axoplasmic organelles was surprising to us as we presumed a random distribution of APP along the organelle surface. In other studies, myosin V antibodies decorated squid axoplasmic organelles throughout the organelle surface [62]. It has been speculated that APP may be associate with lipid rafts as APP is found in abundance in insoluble detergent extracts, a hallmark of lipid raft biochemistry [63–67]. The size of lipid rafts is estimated in the range of 20–40 microns. APP-organelles, in this study, were approximately 100 nm in diameter and are therefore able to accommodate the putative raft structure. Rafts may define the boundary of APP clusters and drive the proximity of APP monomers to one another. Clustering may allow for a coordinated function for APP monomers and rafts may provide a framework for such a coordinated function without requiring direct monomer/monomer interaction. In other studies oligomerization of amyloid beta-protein has been detected in human spinal fluid and within primary human neuron suggesting that aggregation begins intracellularly [68].

Clustering may also contribute to Aβ aggregation and deposition, however whether APP is processed in squid has not been determined. By Western blot of extruded squid axoplasm we failed to detect cleavage products for APP suggesting that this protein is not processed within axoplasmic organelles prior to vesicle fusion with the cell membrane. However, N-terminal fragments of APP (NTFs) are of similar molecular weight as full-length APP and therefore these fragments may not be differentiated in our Western blot experiments. Of ~23,000 expressed sequence tags (ESTs) we previously obtained beta and gamma secretase transcripts were not detected. However, sequences for subunits such as PEN-2 have been found in other mollusks including the sea hare *Aplysia californica*, the freshwater snail *Biomphalaria glabrata*, and the Pacific Oyster *Crassostrea Gigis*. Whether the presence of these transcripts is indicative of APP processing is debatable. However, the function of wildtype APP may be conserved cross species. The clustering of APP to a focal point along the microtubules indicated an orientation to the organelle as the APP microdomain defines a functional surface of the organelle membrane. The fact that the C-terminal of APP is highly conserved demonstrates that the C-terminal is a functional important part of the molecule. That APP is clustered and found at the organelle/microtubule interface leads us to propose that the native function of APP, like Tau has a microtubule-based function and that clustering likely contributes to Aβ deposition.

## References

[1] Brookmeyer R, Johnson E, Ziegler-Graham K, Arrighi HM. Forecasting the global burden of Alzheimer’s disease. Alzheimers Dement. 2008 9;4(5):316–23. 19595937 10.1016/j.jalz.2007.04.381

[2] Kidd M. Paired helical filaments in electron microscopy of Alzheimer’s disease. Nature. 1963 1 12;197:192–3. 10.1038/197192b0 14032480

[3] Roth M, Tomlinson BE, Blessed G. Correlation between scores for dementia and counts of ‘senile plaques’ in cerebral grey matter of elderly subjects. Nature. 1966 1 1;209(5018):109–10. 5927229 10.1038/209109a0

[4] Masters CL, Simms G, Weinman NA, Multhaup G, McDonald BL, Beyreuther K. Amyloid plaque core protein in Alzheimer disease and Down syndrome. Proc Natl Acad Sci U S A. 1985 6;82(12):4245–9. 3159021 10.1073/pnas.82.12.4245 PMC397973

[5] Selkoe DJ. Alzheimer’s disease: genes, protein, and therapy. Physiol Rev. 2001 4;81(2):741–66. Review. 11274343 10.1152/physrev.2001.81.2.741

[6] Glenner GG, Wong CW. Alzheimer’s disease: initial report of the purification and characterization of a novel cerebrovascular amyloid protein. Biochem Biophys Res Commun. 1984 5 16;120(3):885–90. 6375662 10.1016/s0006-291x(84)80190-4

[7] Goate A, Chartier-Harlin MC, Mullan M, Brown J, Crawford F, Fidani L, Giuffra L, et al Segregation of a missense mutation in the amyloid precursor protein gene with familial Alzheimer’s disease. Nature. 1991 2 21;349(6311):704–6. 1671712 10.1038/349704a0

[8] Chartier-Harlin MC, Crawford F, Houlden H, Warren A, Hughes D, Fidani L, Goate A, et al Early-onset Alzheimer’s disease caused by mutations at codon 717 of the beta-amyloid precursor protein gene. Nature. 1991 10 31;353(6347):844–6. 1944558 10.1038/353844a0

[9] Crawford F, Hardy J, Mullan M, Goate A, Hughes D, Fidani L, et al Sequencing of exons 16 and 17 of the beta-amyloid precursor protein gene in 14 families with early onset Alzheimer’s disease fails to reveal mutations in the beta-amyloid sequence. Neurosci Lett. 1991 11 25;133(1):1–2. 1791986 10.1016/0304-3940(91)90042-r

[10] Thonberg H, Fallström M, Björkström J, Schoumans J, Nennesmo I, Graff C. Mutation screening of patients with Alzheimer disease identifies APP locus duplication in a Swedish patient. BMC Res Notes. 2011 11 1;4:476 10.1186/1756-0500-4-476 22044463 PMC3216298

[11] Kang J, Lemaire HG, Unterbeck A, Salbaum JM, Masters CL, Grzeschik KH, et al The precursor of Alzheimer’s disease amyloid A4 protein resembles a cell-surface receptor. Nature. 1987 2 19–25;325(6106):733–6. 2881207 10.1038/325733a0

[12] Lichtenthaler SF. Alpha-secretase cleavage of the amyloid precursor protein: proteolysis regulated by signaling pathways and protein trafficking. Curr Alzheimer Res. 2012 2;9(2):165–77. 21605033 10.2174/156720512799361655

[13] Dyrks T, Weidemann A, Multhaup G, Salbaum JM, Lemaire HG, Kang J, Müller-Hill B, et al Identification, transmembrane orientation and biogenesis of the amyloid A4 precursor of Alzheimer’s disease. EMBO J. 1988 4;7(4):949–57. 2900137 10.1002/j.1460-2075.1988.tb02900.x PMC454420

[14] Muresan V, Varvel NH, Lamb BT, Muresan Z. The cleavage products of amyloid-beta precursor protein are sorted to distinct carrier vesicles that are independently transported within neurites. J Neurosci. 2009 3 18;29(11):3565–78. 10.1523/JNEUROSCI.2558-08.2009 19295161 PMC2669751

[15] Kamal A, Stokin GB, Yang Z, Xia CH, Goldstein LS. Axonal transport of amyloid precursor protein is mediated by direct binding to the kinesin light chain subunit of kinesin-1. Neuron. 2000 11;28(2):449–59. 11144355 10.1016/s0896-6273(00)00124-0

[16] Lazarov O, Morfini GA, Lee EB, Farah MH, Szodorai A, DeBoer SR, et al Axonal transport, amyloid precursor protein, kinesin-1, and the processing apparatus: revisited. J Neurosci. 2005 3 2;25(9):2386–95. 15745965 10.1523/JNEUROSCI.3089-04.2005 PMC6726084

[17] Brady ST, Lasek RJ, Allen RD. Fast axonal transport in extruded axoplasm from squid giant axon. Science. 1982 12 10;218(4577):1129–31. 6183745 10.1126/science.6183745

[18] Allen RD, Metuzals J, Tasaki I, Brady ST, Gilbert SP. Fast axonal transport in squid giant axon. Science. 1982 12 10;218(4577):1127–9. 6183744 10.1126/science.6183744

[19] Vale RD, Schnapp BJ, Mitchison T, Steuer E, Reese TS, Sheetz MP. Different axoplasmic proteins generate movement in opposite directions along microtubules in vitro. Cell. 1985 12;43(3 Pt 2):623–32. 2416467 10.1016/0092-8674(85)90234-x

[20] Vale RD, Reese TS, Sheetz MP. Identification of a novel force-generating protein, kinesin, involved in microtubule-based motility. Cell. 1985 12;43(3 Pt 2):623–32. 3926325 10.1016/s0092-8674(85)80099-4 PMC2851632

[21] DeGiorgis JA, Cavaliere KR, Burbach JP. Identification if molecular motors in the Woods Hole squid, Loligo pealei: an expressed sequence tag approach. Cytoskeleton (Hoboken). 2011 10;68(10):566–77. 10.1002/cm.20531 21913340

[22] Satpute-Krishnan P, DeGiorgis JA, Conley MP, Jang M, Bearer EL. A peptide zipcode sufficient for anterograde transport within amyloid precursor protein. Proc Natl Acad Sci U S A. 2006 10 31;103(44):16532–7. 17062754 10.1073/pnas.0607527103 PMC1621108

[23] Schnapp BJ, Vale RD, Sheetz MP, Reese TS. Single microtubules from squid axoplasm support bidirectional movement of organelles. Cell. 1985 2;40(2):455–62. 2578325 10.1016/0092-8674(85)90160-6

[24] Vale RD, Schnapp BJ, Reese TS, Sheetz MP. Movement of organelles along filaments dissociated from the axoplasm of the squid giant axon. Cell. 1985 2;40(2):449–54. 2578324 10.1016/0092-8674(85)90159-x

[25] Schnapp BJ, Reese TS, Bechtold R. Kinesin is bound with high affinity to squid axon organelles that move to the plus-end of microtubules. J Cell Biol. 1992 10;119(2):389–99. 1400582 10.1083/jcb.119.2.389 PMC2289649

[26] DeGiorgis JA, Petukhova TA, Evans TA, Reese TS. Kinesin-3 is an organelle motor in the squid giant axon. Traffic. 2008 11;9(11):1867–77. 10.1111/j.1600-0854.2008.00809.x Epub 2008 Aug 4. 18928504

[27] Paschal BM, Shpetner HS, Vallee RB. MAP 1C is a microtubule-activated ATPase which translocates microtubules in vitro and has dynein-like properties. J Cell Biol. 1987 9;105(3):1273–82. 2958482 10.1083/jcb.105.3.1273 PMC2114794

[28] Schnapp BJ, Reese TS. Dynein is the motor for retrograde axonal transport of organelles. Proc Natl Acad Sci U S A. 1989 3;86(5):1548–52. 2466291 10.1073/pnas.86.5.1548 PMC286735

[29] Kuznetsov SA, Gelfand VI. Purification of kinesin from the brain. Methods Mol Biol. 2001;164:1–7. 11217600 10.1385/1-59259-069-1:1

[30] DeLuca JG, Newton CN, Himes RH, Jordan MA, Wilson L. Purification and characterization of native conventional kinesin, HSET, and CENP-E from mitotic hela cells. J Biol Chem. 2001 7 27;276(30):28014–21. 11382767 10.1074/jbc.M102801200

[31] Sigua R, Tripathy S, Anand P, Gross SP. Isolation and purification of kinesin from Drosophila embryos. J Vis Exp. 2014 5 6;(87). 10.3791/3501 PMC3466667 22565641

[32] Wasco W, Bupp K, Magendantz M, Gusella JF, Tanzi RE and Solomon F. Identification of a mouse brain cDNA that encodes a protein related to the Alzheimer disease-associated amyloid beta protein precursor. Proc Natl Acad Sci U S A. 1992 11 15;89(22):10758–62. 1279693 10.1073/pnas.89.22.10758 PMC50421

[33] Wasco W, Gurubhagavatula S, Paradis MD, Romano DM, Sisodia SS, Hyman BT, et al Isolation and characterization of APLP2 encoding a homologue of the Alzheimer’s associated amyloid beta protein precursor. Nat Genet. 1993 9;5(1):95–100. 8220435 10.1038/ng0993-95

[34] Paliga K, Peraus G, Kreger S, Dürrwang U, Hesse L, Multhaup G, et al Human amyloid precursor-like protein 1—cDNA cloning, ectopic expression in COS-7 cells and identification of soluble forms in the cerebrospinal fluid. Eur J Biochem. 1997 12 1;250(2):354–63. 9428684 10.1111/j.1432-1033.1997.0354a.x

[35] Eggert S, Paliga K, Soba P, Evin G, Masters CL, Weidemann A, et al The proteolytic processing of the amyloid precursor protein gene family members APLP-1 and APLP-2 involves alpha-, beta-, gamma-, and epsilon-like cleavages: modulation of APLP-1 processing by n-glycosylation. J Biol Chem. 2004 4 30;279(18):18146–56. 14970212 10.1074/jbc.M311601200

[36] Zheng H, Jiang M, Trumbauer ME, Sirinathsinghji DJ, Hopkins R, Smith DW, et al beta-Amyloid precursor protein-deficient mice show reactive gliosis and decreased locomotor activity. Cell. 1995 5 19;81(4):525–31. 7758106 10.1016/0092-8674(95)90073-x

[37] von Koch CS, Zheng H, Chen H, Trumbauer M, Thinakaran G, vander Ploeg LH, Price DL, et al (1997) Generation of APLP2 KO mice and early postnatal lethality in APLP2/APP double KO mice. Neurobiol Aging. 1997 Nov-Dec;18(6):661–9. 9461064 10.1016/s0197-4580(97)00151-6

[38] Heber S, Herms J, Gajic V, Hainfellner J, Aguzzi A, Rülicke T, et al Mice with combined gene knock-outs reveal essential and partially redundant functions of amyloid precursor protein family members. J Neurosci. 2000 11 1;20(21):7951–63. 11050115 10.1523/JNEUROSCI.20-21-07951.2000 PMC6772747

[39] Liu X, Yu X, Zack DJ, Zhu H and Qian J. TiGER: a database for tissue-specific gene expression and regulation. BMC Bioinformatics. 2008 6 9;9:271 10.1186/1471-2105-9-271 18541026 PMC2438328

[40] Vega JA, Diaz-Trelles R, Haro JJ, del Valle ME, Naves FJ, Fernández-Sánchez MT. Beta-amyloid precursor protein in human digital skin. Neurosci Lett. 1995 6 9;192(2):132–6. 7675321 10.1016/0304-3940(95)11618-7

[41] Shigematsu K, McGeer PL, McGeer EG. Localization of amyloid precursor protein in selective postsynaptic densities of rat cortical neurons. Brain Res. 1992 10 2;592(1–2):353–7. 1280522 10.1016/0006-8993(92)91697-d

[42] Koo EH, Squazzo SL, Selkoe DJ and Koo CH. Trafficking of cell-surface amyloid beta-protein precursor. I. Secretion, endocytosis and recycling as detected by labeled monoclonal antibody. J Cell Sci. 1996 5;109 (Pt 5):991–8. 8743946 10.1242/jcs.109.5.991

[43] Yamazaki T, Koo EH and Selkoe DJ. Trafficking of cell surface amyloid beta-protein precursor. II. Endocytosis, recycling and lysosomal targeting detected by immunolocalization. J Cell Sci. 1996 5;109 (Pt 5):999–1008. 8743947 10.1242/jcs.109.5.999

[44] Haass C, Koo EH, Mellon A, Hung AY and Selkoe DJ. Targeting of cell-surface beta-amyloid precursor protein to lysosomes: alternative processing into amyloid-bearing fragments. Nature. 1992 6 11;357(6378):500–3. 1608449 10.1038/357500a0

[45] Villegas C, Muresan V, Ladescu Muresan Z. Dual-tagged amyloid-β precursor protein reveals distinct transport pathways of its N- and C-terminal fragments. Hum Mol Genet. 2014 3 15;23(6):1631–43. 10.1093/hmg/ddt555 24203698 PMC3929097

[46] Groemer TW, Thiel CS, Holt M, Riedel D, Hua Y, Hüve J, Wilhelm BG, et al Amyloid precursor protein is trafficked and secreted via synaptic vesicles. PLoS One. 2011 4 27;6(4):e18754 10.1371/journal.pone.0018754 21556148 PMC3083403

[47] Fanutza T, Del Prete D, Ford MJ, Castillo PE, D'Adamio L. APP and APLP2 interact with the synaptic release machinery and facilitate transmitter release at hippocampal synapses. Elife. 2015 11 9;4 pii: e09743 10.7554/eLife.09743 26551565 PMC4755753

[48] Caillé I, Allinquant B, Dupont E, Bouillot C, Langer A, Müller U, Prochiantz A. Soluble form of amyloid precursor protein regulates proliferation of progenitors in the adult subventricular zone. Development. 2004 5;131(9):2173–81. 15073156 10.1242/dev.01103

[49] Wang Z, Wang B, Yang L, Guo Q, Aithmitti N, Songyang Z, et al Presynaptic and postsynaptic interaction of the amyloid precursor protein promotes peripheral and central synaptogenesis. J Neurosci. 2009 9 2;29(35):10788–801. 10.1523/JNEUROSCI.2132-09.2009 19726636 PMC2757256

[50] Seabrook GR, Smith DW, Bowery BJ, Easter A, Reynolds T, Fitzjohn SM, et al Mechanisms contributing to the deficits in hippocampal synaptic plasticity in mice lacking amyloid precursor protein. Neuropharmacology. 1999 3;38(3):349–59. 10219973 10.1016/s0028-3908(98)00204-4

[51] Mileusnic R, Lancashire CL, Rose SP. Amyloid precursor protein: from synaptic plasticity to Alzheimer's disease. Ann N Y Acad Sci. 2005 6;1048:149–65. 16154929 10.1196/annals.1342.014

[52] Palacios G, Mengod G, Tortosa A, Ferrer I, Palacios JM. Increased beta-amyloid precursor protein expression in astrocytes in the gerbil hippocampus following ischaemia: association with proliferation of astrocytes. Eur J Neurosci. 1995 3 1;7(3):501–10. 7773447 10.1111/j.1460-9568.1995.tb00346.x

[53] Breen KC, Bruce M, Anderton BH. Beta amyloid precursor protein mediates neuronal cell-cell and cell-surface adhesion. J Neurosci Res. 1991 1;28(1):90–100. 1645774 10.1002/jnr.490280109

[54] Coburger I, Dahms SO, Roeser D, Gührs KH, Hortschansky P, Than ME. Analysis of the overall structure of the multi-domain amyloid precursor protein (APP). PLoS One. 2013 12 4;8(12):e81926 10.1371/journal.pone.0081926 24324731 PMC3852973

[55] Barnham KJ, McKinstry WJ, Multhaup G, Galatis D, Morton CJ, Curtain CC, et al Structure of the Alzheimer's disease amyloid precursor protein copper binding domain. A regulator of neuronal copper homeostasis. J Biol Chem. 2003 5 9;278(19):17401–7. 12611883 10.1074/jbc.M300629200

[56] Owen DJ, Collins BM. Vesicle transport: a new player in APP trafficking. Curr Biol. 2010 5 11;20(9):R413–5. 10.1016/j.cub.2010.03.017 20462485

[57] Gunawardena S, Yang G, Goldstein LS. Presenilin controls kinesin-1 and dynein function during APP-vesicle transport in vivo. Hum Mol Genet. 2013 10 1;22(19):3828–43. 10.1093/hmg/ddt237 23710041 PMC3766177

[58] Fu MM, Holzbaur EL. JIP1 regulates the directionality of APP axonal transport by coordinating kinesin and dynein motors. J Cell Biol. 2013 8 5;202(3):495–508. 10.1083/jcb.201302078 23897889 PMC3734084

[59] Brunholz S, Sisodia S, Lorenzo A, Deyts C, Kins S, Morfini G. Axonal transport of APP and the spatial regulation of APP cleavage and function in neuronal cells. Exp Brain Res. 2012 4;217(3–4):353–64. 10.1007/s00221-011-2870-1 21960299 PMC3670699

[60] Koo EH, Sisodia SS, Archer DR, Martin LJ, Weidemann A, Beyreuther K, et al Precursor of amyloid protein in Alzheimer disease undergoes fast anterograde axonal transport. Proc Natl Acad Sci U S A. 1990 2;87(4):1561–5. 1689489 10.1073/pnas.87.4.1561 PMC53515

[61] Gunawardena S, Goldstein LS. Disruption of axonal transport and neuronal viability by amyloid precursor protein mutations in Drosophila. Neuron. 2001 11 8;32(3):389–401. 11709151 10.1016/s0896-6273(01)00496-2

[62] Tabb JS, Molyneaux BJ, Cohen DL, Kuznetsov SA, Langford GM. Transport of ER vesicles on actin filaments in neurons by myosin V. J Cell Sci. 1998 11;111 (Pt 21):3221–34. 9763516 10.1242/jcs.111.21.3221

[63] Karnovsky MJ, Kleinfeld AM, Hoover RL, Klausner RD. The concept of lipid domains in membranes. J Cell Biol. 1982 7;94(1):1–6. 6889603 10.1083/jcb.94.1.1 PMC2112185

[64] Simons K, Ikonen E. Functional rafts in cell membranes. Nature. 1997 6 5;387(6633):569–72. 9177342 10.1038/42408

[65] Simons M, Keller P, De Strooper B, Beyreuther K, Dotti CG, Simons K. Cholesterol depletion inhibits the generation of beta-amyloid in hippocampal neurons. Proc Natl Acad Sci U S A. 1998 5 26;95(11):6460–4. 9600988 10.1073/pnas.95.11.6460 PMC27798

[66] Brown DA, London E. (1997) Structure of detergent-resistant membrane domains: does phase separation occur in biological membranes? Biochem Biophys Res Commun. 1997 11 7;240(1):1–7. Review. 9367871 10.1006/bbrc.1997.7575

[67] Ehehalt R, Keller P, Haass C, Thiele C, Simons K. Amyloidogenic processing of Alzheimer beta-amyloid precursor protein depends on lipid rafts. J Cell Biol. 2003 1 6;160(1):113–23. 12515826 10.1083/jcb.200207113 PMC2172747

[68] Walsh DM, Tseng BP, Rydel RE, Podlisny MB, Selkoe DJ. The oligomerization of amyloid beta-protein begins intracellularly in cells derived from human brain. Biochemistry. 2000 9 5;39(35):10831–9. 10978169 10.1021/bi001048s

